# Influence of leader humility on bootlegging innovative behavior—a serial mediation model of work attention and thriving at work

**DOI:** 10.3389/fpsyg.2026.1775820

**Published:** 2026-03-02

**Authors:** Junzhu Zhang, MyeongCheol Choi, WonGyu Lee, Hann Earl Kim

**Affiliations:** 1 Department of Business Management, Gachon University, Seongnam, Republic of Korea; 2 Department of Intellectual Property Convergence, Gyeongsang National University, Jinju, Republic of Korea

**Keywords:** bootlegging innovative behavior, leader humility, serial mediation model, thriving at work, work attention

## Abstract

Bootlegging innovative behavior—a form of self-initiated, unauthorized innovation—is critical for maintaining organizational adaptability and competitiveness. Drawing upon Conservation of Resources (COR) theory and Self-Determination Theory (SDT), this study investigates how leader humility influences employees’ bootlegging innovative behavior, examining the sequential mediating roles of work attention and thriving at work. Data were gathered from 427 employees across manufacturing and service enterprises in eastern China. Analyses conducted via SPSS, Stata, and M-plus revealed that leader humility exerts a significant direct effect on bootlegging innovative behavior. Furthermore, both work attention and thriving at work functioned as significant mediators. We validated a serial mediation pathway whereby leader humility enhances work attention, which in turn fosters thriving at work, ultimately leading to increased bootlegging innovative behavior. By acknowledging their limitations and valuing employee contributions, humble leaders foster psychological safety and cognitive focus. This allows employees to redirect attentional resources toward meaningful tasks, promoting a state of cognitive engagement. This engagement enhances learning and vitality—the core components of thriving at work—which subsequently stimulates the intrinsic motivation necessary for such informal, self-driven innovation. These findings extend the theoretical understanding of how leader humility cultivates bottom-up innovation, highlighting the crucial cognitive and psychological mechanisms that underpin bootlegging. Practically, this study underscores that developing leader humility and fostering a thriving workplace are viable strategies for encouraging responsible innovation within organizations.

## Introduction

Organizations increasingly operate in complex and uncertain market environments (Wen et al., 2021). This context exposes the limitations of traditional, top-down research and development (R&D) processes. While these formal structures aid strategic control, their inherent rigidity and slow responsiveness often hinder adaptation to dynamic market demands. The limitations of formal channels have shifted research focus toward informal innovation. Attention has turned to employee-driven initiatives that originate spontaneously, often without formal authorization or managerial direction (Kesting and Ulhøi, 2010; Pahos et al., 2025).

“Bootlegging innovative behavior” (BIB) has become a central topic within this domain (Criscuolo et al., 2014). The term refers to employees’ covert, unauthorized engagement in innovation projects, often using personal time or discretionary organizational resources (Criscuolo et al., 2014; Globocnik and Salomo, 2015). Bootlegging innovative behavior is an informal form of innovation with inherent risk. Unlike formally authorized innovation, it unfolds outside official organizational channels. It often lacks direct managerial approval and dedicated resources (Globocnik and Salomo, 2015). This dual nature makes bootlegging conceptually ambiguous. Such behavior is inherently paradoxical. It can represent a form of “positive deviance” that challenges bureaucratic norms to achieve breakthroughs (Kesting and Ulhøi, 2010; Slaughter et al., 2020). Yet, it also exposes the individual to significant risks, including career sanctions for violating rules or misusing resources (Kesting and Ulhøi, 2010; Globocnik and Salomo, 2015; Wang et al., 2025). This tension sets bootlegging apart from more traditional leadership–innovation phenomena. It raises a key question. Under what conditions does bootlegging remain constructive rather than dysfunctional? Addressing this question requires attention to leadership context. Leaders play a central role in shaping how employees interpret risk, autonomy, and experimentation (Lyu et al., 2022; Wang et al., 2025).

It is important to note that not all bootlegging innovative behavior leads to positive outcomes for individuals or organizations. Based on the perspective of constructive deviance, we distinguish between constructive bootlegging innovative behavior and dysfunctional or high-risk bootlegging innovative behavior. Constructive bootlegging innovative behavior is typically driven by prosocial motives and a learning orientation. It may eventually be incorporated into formal innovation processes (Ding et al., 2023; Shang, 2024; Zhang et al., 2025). This form of bootlegging is oriented toward long-term value creation and organizational learning (Globocnik et al., 2022). In contrast, high-risk bootlegging innovative behavior may involve resource misuse or misalignment with organizational goals. Such behavior is likely to undermine organizational trust and performance (Globocnik et al., 2022; Ding et al., 2023). Bootlegging innovative behavior is inherently ambiguous. Recognizing this tension helps distinguish bootlegging from conventional innovation and clarifies why leadership context is critical.

Prior research shows that bootlegging innovative behavior is more likely to produce positive outcomes under specific organizational conditions. These conditions include a learning-oriented climate, tolerance for experimentation, and high psychological safety (Globocnik et al., 2022; Lyu et al., 2022; Wang et al., 2025). Leader humility helps shape such conditions by legitimizing exploratory behavior and reducing fear of failure (Qu et al., 2023). This context increases the likelihood that bootlegging innovative behavior remains constructive. Therefore, we define bootlegging innovative behavior as a learning-oriented, bottom-up innovation activity that emerges under supportive leadership contexts.

Organizational context, particularly leadership style, profoundly influences employees’ constructive innovative behaviors (Frazier et al., 2017). Among various leadership approaches, leader humility has garnered significant scholarly attention. It is defined as a positive, relational leadership style characterized by accurate self-awareness, an appreciation of others’ strengths, and a teachable orientation (Owens et al., 2013; Luo et al., 2022). Humble leaders foster a climate of psychological safety and trust. They achieve this by acknowledging personal limitations, communicating non-defensively, and genuinely valuing employee contributions (Wang et al., 2018). Such safety is a critical precondition for employees to engage in unauthorized innovation (Wang et al., 2018; Elhadidy and Gao, 2025). Establishing a direct link between humble leadership and bootlegging is insufficient. The critical question concerns how this positive leadership style translates into concrete employee innovation (Owens and Hekman, 2016; Luo et al., 2022; Wang et al., 2024).

To address the research question, this study draws on multiple theoretical perspectives with clear roles. Conservation of resources theory serves as the core framework. It explains how leader humility shapes employees’ allocation of cognitive resources, especially attention. Self-determination theory and attention-based views play a complementary role. They clarify how preserved attentional resources support need satisfaction, vitality, and learning. Positive organizational psychology provides a broader context for understanding thriving at work as a dynamic psychological state. These perspectives are not treated as parallel explanations. They are integrated to capture distinct but connected stages of the mediation process.

This study draws upon Conservation of Resources (COR) Theory (Hobfoll, 1989). We propose that humble leadership reduces an employee’s depletion of psychological resources, especially those spent on defensive posturing. This preservation of resources frees limited cognitive capacities, such as attention. These freed resources can then be reinvested in focused work engagement and psychological growth (Halbesleben et al., 2014). In complex organizational environments, attention is a scarce yet critical cognitive resource (Kahneman, 1973). How employees allocate this resource determines not only their task performance but also their capacity for creative problem-solving (Wang et al., 2018). When psychological safety is low, individuals expend significant cognitive resources on self-protection and impression management. This diversion of resources detracts from core task engagement (Edmondson, 1999). Humble leaders, through their openness and supportive behaviors, can alleviate this anxiety and cognitive drain. Employees are then able to channel greater attention toward core tasks and problem diagnosis. This focused state is essential for deep cognitive engagement and, consequently, for innovation (Wang et al., 2018).

Sustained attentional investment does more than improve immediate performance; it can cultivate the positive psychological state of thriving at work (Spreitzer et al., 2005; Iqbal et al., 2020). Thriving at work is defined as the joint experience of vitality (feeling energized) and learning (growing; Porath et al., 2012). It functions as a psychological engine for personal development and sustained innovation (Porath et al., 2012; Huang et al., 2022). According to Self-Determination Theory (Deci and Ryan, 2000), thriving helps fulfill basic psychological needs for autonomy, competence, and relatedness. The fulfillment of these needs, in turn, strengthens intrinsic motivation and innovative self-efficacy (Wang et al., 2024). Psychology and neuroscience confirm that attention is a core mechanism for high performance and creativity (Rueda et al., 2015; Benedek and Fink, 2019). Deep focus, or the “flow” state, is central to this process. Neuroimaging evidence reveals that during flow states, brain regions linked to high-level cognitive control, such as the prefrontal cortex, show heightened activation (Van den Broeck et al., 2016). Concurrently, activity in the brain’s default mode network is suppressed (Ulrich et al., 2016). This neural pattern significantly enhances cognitive control and task enjoyment (Ulrich et al., 2016). This optimal state is biochemically linked to dopaminergic–noradrenergic synergy. This system sustains alertness and pleasure during challenges, which reinforces learning and innovative tendencies (Nakamura and Csikszentmihalyi, 2014). Humble leadership provides ideal external conditions for this to occur. By reducing social comparison and the fear of failure, humble leaders help employees enter this focused cognitive-affective state. Research on leader humility and innovation has grown, a key limitation persists. Most studies have focused on formal creativity or innovation performance (Ye et al., 2020; Tariq et al., 2023). The mechanisms through which this leadership style fosters informal or deviant innovation, such as bootlegging, remain poorly elucidated. Bootlegging entails higher autonomy and greater personal risk than conventional innovation (Criscuolo et al., 2014; Globocnik, 2023). These distinct characteristics demand dedicated theoretical exploration of its underlying cognitive and psychological pathways.

Previous studies have often adopted single-mediator perspectives, focusing on concepts like psychological safety or learning climate (Carmeli et al., 2015). This approach tends to overlook the dynamic interplay between cognitive and psychological resources at the individual level (Alomani et al., 2022). In practice, employee innovation rarely stems directly from affective states alone; it is fundamentally grounded in attentional allocation and the progressive accumulation of psychological resources (Iqbal et al., 2020). This study incorporates work attention and thriving at work as sequential mediators. We propose a cognitive-psychological chain model to explicate the mechanisms through which humble leadership fosters bootlegging innovation. While psychology and neuroscience have long established the neural basis of focus and learning (Posner & Rothbart, 2007), organizational behavior studies have yet to fully integrate leadership styles with these cognitive mechanisms. Conceptualizing attention as a form of “cognitive capital” offers a richer understanding of how leadership behaviors influence innovation through resource conservation and transformation (Alomani et al., 2022). Based on Conservation of Resources Theory and Self-determination Theory, we argue that attentional resources serve as a key cognitive channel. Through this channel, basic psychological needs can be fulfilled. Humble leaders, through self-disclosure, inclusiveness, and empowerment (Owens et al., 2013), alleviate employees’ cognitive load. Freed from the need to expend resources on psychological defense, employees can dedicate greater focus to core tasks and problem-solving. In addition, leader humility protects employees’ attentional resources. This protection allows employees to allocate attention more autonomously. It also reduces perceived interpersonal threat (Rego et al., 2017; Hadmar et al., 2022). This process not only enhances learning but also strengthens vitality and intrinsic motivation (Ryan and Deci, 2000).

Building on this perspective, we posit that leader humility influences bootlegging through two distinct pathways. The first is a cognitive resource pathway. Humble leadership enhances employee work attention, enabling them to concentrate finite cognitive resources on complex problem-solving and idea exploration. The second is a psychological resource pathway. This leadership style fosters thriving at work, which in turn stimulates intrinsic motivation and innovation resilience (Spreitzer et al., 2005). The interaction of these mechanisms suggests a chained mediation effect. This chain may empower employees to persist in unauthorized innovation, even under conditions of uncertainty. Our study integrates these cognitive and psychological perspectives. We construct and test a sequential mediation model: Leader Humility → Work Attention → Thriving at Work → Bootlegging Innovative Behavior. This model addresses several key questions. We investigate whether leader humility significantly enhances employee bootlegging. We also examine if work attention functions as a core cognitive mediator, and whether thriving at work serves as a subsequent psychological mechanism. The central aim is to test if these mechanisms operate sequentially as a cognitive-psychological chain. This study addresses the “how” and “why” questions central to the humble leadership–innovation relationship (Owens et al., 2013). By incorporating work attention and thriving at work, we extend leadership research into the domains of neurocognitive function and psychological energy. This approach also fosters interdisciplinary integration between leadership studies and cognitive science. The findings offer clear practical implications. Organizations can benefit from cultivating humble leadership behaviors. These include encouraging self-reflection, appreciating others’ strengths, and fostering experimental tolerance. Such actions help build a psychologically safe environment, which is essential for innovation. Managers should also attend to the cognitive and psychological energy levels of their employees. Specific tools, such as thoughtful job design, constructive feedback, and empowerment, can sustain employee focus. These practices nurture the enthusiasm for learning and ultimately unlock the potential for informal innovation.

## Theory and hypotheses

### Leader humility and bootlegging innovation behavior

Humility is defined as a relatively stable trait that is rooted in a certain view of the self, i.e., that there is something greater than the self (Morris et al., 2005; Owens et al., 2013). Humble people do not have a strong need to dominate others or seek self-improvement (Morris et al., 2005); they are aware of their own shortcomings and strengths (Owens et al., 2013), and can appreciate others’ contributions and perspectives (Owens and Hekman, 2012). Humility is often considered a character strength, closely related to the interdependence of today’s organizations and markets, and uniquely representative (Owens and Hekman, 2012). Humility brings with it a perspective of pro-social relationships that is increasingly necessary to work with different parties within and across organizational boundaries (Owens and Hekman, 2012). Humility has been shown to be a source of competitive advantage for individuals, leaders, and organizations (Owens and Hekman, 2012; Wang et al., 2018). Humble organizational leaders set an example for today’s dynamic work environment, which emphasizes growth and continuous learning (Owens and Hekman, 2012).

Nielsen and Marrone (Owens and Hekman, 2012) argued that humility is a relationship between self and others, involving internal self-regulation and the ability to promote the formation of prosocial relationships. Additionally, previous scholars have explored the behavior and attribution of humility in social contexts, emphasizing the relational and interpersonal aspects of humility. Regarding humility relationships, phenotypic humility is defined as an interpersonal trait that manifests itself in a social context, implying a willingness to see oneself accurately, appreciating the strengths and contributions of others and teachability (Owens et al., 2013). Regarding interpersonal humility, relational humility is defined as not being self-centered but being other-oriented in interpersonal interactions, with an accurate view of the self (Owens et al., 2013; Exline et al., 2011). Humble leaders are often genuinely appreciative of employees’ contributions and strengths, open to new ideas and information, and open to feedback from others on their actions and perspectives (Owens et al., 2013; Owens and Hekman, 2012).

Under such leadership styles, employees experience a sense of psychological safety and an atmosphere of trust, which reduces their fear of making mistakes or facing failure (Edmondson, 1999; Exline et al., 2011). Consequently, they become more inclined to engage in innovative exploration without formal authorization—commonly referred to as “bootlegging innovative behavior” (Criscuolo et al., 2014). This term describes employees’ self-initiated innovation activities that are carried out secretly, without official approval or access to organizational resources, driven by their intrinsic motivation to promote improvement and learning (Criscuolo et al., 2014; Qu et al., 2023).

Different traits will have different influences on employees, and leaders’ decisions, perceptions, words and deeds may all be influencing factors for employees’ innovative behaviors (Wang et al., 2018). Unlike formal innovation, bootlegging innovative behavior represents an informal, potentially deviant, yet positively oriented approach to innovation (Criscuolo et al., 2014; Slaughter et al., 2020). It describes the process by which employees spontaneously identify problems, generate ideas, and pursue experimentation and improvements, often in the absence of explicit resources or support (Qu et al., 2023).

While it shares core components with traditional innovation—such as idea generation, testing, and implementation—its defining features are the clandestine nature and high autonomy of these activities (Slaughter et al., 2020). Creativity remains a necessary antecedent for such behavior (Amabile, 1996). However, departing from traditional innovation, bootlegging often manifests as “positive deviance,” wherein employees proactively bypass institutional constraints to achieve work improvements (Criscuolo et al., 2014). Although this may be perceived as a procedural violation in the short term, it can significantly foster organizational learning and adaptability in the long run (Qu et al., 2023).

Research indicates that factors within the organizational environment, particularly psychological safety and leadership behaviors, significantly influence an employee’s decision to engage in bootlegging (Edmondson, 1999; Wang et al., 2018). Leader humility, for instance, fosters a sense of safety through open dialog and non-punitive feedback, making employees more inclined to validate novel ideas covertly (Owens and Hekman, 2012; Wang et al., 2018). Furthermore, leader humility can bolster an atmosphere of team empowerment and cooperation, which is conducive to informal learning and the creation of shared experimental spaces (Owens and Hekman, 2012; Wang et al., 2018).

Drawing from Cognitive Evaluation Theory (Deci and Ryan, 2000), when individuals perceive their environment as supportive and respectful, their intrinsic motivation to sustain challenging activities is enhanced. In high-trust contexts, this motivation is particularly likely to translate into bootlegging innovative behavior (Criscuolo et al., 2014; Qu et al., 2023). Similarly, Social Learning Theory (Bandura, 1977) suggests that leader humility acts as a role model, demonstrating openness and learning-oriented behaviors. Through observation and imitation, employees are likely to develop a similar initiative and experimental spirit (Owens and Hekman, 2012; Wang et al., 2018). This social learning mechanism encourages employees to seek innovative opportunities, both within and beyond organizational boundaries, and to pursue them even without formal approval. Leader humility not only fosters psychological growth but also offers guidance and support when employees encounter difficulties (Owens and Hekman, 2012; Wang et al., 2018), thereby strengthening their tolerance for the risks inherent in innovation.

Therefore, by cultivating a supportive atmosphere, enhancing psychological safety, and promoting task focus, leader humility enables employees to spontaneously engage in bootlegging innovative behavior under conditions of perceived low risk. This, in turn, drives the organization’s long-term adaptation and evolution (Qu et al., 2023). In summary, this study posits that leader humility is a positive leadership trait. Through the psychological and social mechanisms detailed above, its associated characteristics and behavioral patterns are argued to foster employee bootlegging innovative behavior.

*H*1: Leader humility positively influences employees’ bootlegging innovative behavior.

### Mediating effect of work attention

Some scholars divide work engagement into two core dimensions: work attention and work absorption (Bakker and Demerouti, 2008; Karanika-Murray et al., 2015). Attention is formally defined as cognitive availability and time investment allocated to role-related contemplation (Rothbard and Patil, 2011). Absorption, conceptualized as role immersion intensity (Bakker and Demerouti, 2008; Karanika-Murray et al., 2015), represents an intrinsically motivated state that operates independently of positive affect (Schaufeli et al., 2019). Contemporary neuroscience frameworks characterize attention as either a finite in-formation-processing resource (Kahneman, 1973) or a selective prioritization mechanism (Desimone and Duncan, 1995; Knudsen, 2007). Attentional processes, conceptualized as goal-directed mechanisms encompassing task selection, interference protection, and outcome monitoring (Desimone and Duncan, 1995; Knudsen, 2007; Botvinick and Braver, 2015), operate through controlled governance when aligned with current objectives, wherein cognitive prioritization occurs through hierarchical goal-congruent parameters (Knudsen, 2007). Building upon this conceptual framework, Conservation of Resources theory (Hobfoll, 1989) posits that resource scarcity induces motivational depletion while resource availability facilitates goal attainment, a premise extending to organizational contexts where leader humility manifests as critical psychological resources through encouragement and respect (Qu et al., 2023).

According to previous research, leader humility is fundamental in driving action and cooperation with social bonds (Wang et al., 2018). This leadership style establishes a resource-replenishment mechanism that alleviates employees’ depletion of attentional resources while simultaneously enhancing the value of those resources (Qu et al., 2023). In this process, work attention plays a pivotal mediating role. Specifically, humble leaders foster an environment of high psychological safety by acknowledging their own limitations and openly appreciating the unique contributions of their employees (Qu et al., 2023). In this cognitive state, employees exhibit a heightened propensity to concentrate on task execution rather than interpersonal defensiveness or organizational politics, thereby optimizing task-oriented attentional allocation. When leaders publicly acknowledge employees’ unique contributions and foster knowledge-sharing practices, it cultivates employees’ recognition of their instrumental role in organizational objectives, with such goal alignment reinforcing task motivation and strategic attentional deployment (Rothbard and Patil, 2011). Humble leaders intrinsically motivate employees to engage in work through problem-solving and exploratory cognitive processes, sustaining their cognitive activation. The work attention resources “protected” and “unleashed” through leader humility enable employees to reallocate their conserved cognitive capital, thereby strengthening their bootlegging innovative behavior. This form of behavior represents a bottom-up, unauthorized attempt at innovation that is inherently characterized by high levels of risk and cognitive demand (Criscuolo et al., 2014).

Employees with sufficient attentional resources demonstrate enhanced capacity to synthesize multisource information, diagnose problem etiology, and generate innovative solutions (Lotz and Kristensen, 2012). These are precisely the prerequisites for identifying and executing bootlegging innovative opportunities. Furthermore, sustained attentional engagement enhances persistent cognitive capacity and adaptive problem-solving flexibility in complex tasks, facilitating the production of original yet pragmatic ideas (Botvinick and Braver, 2015; Diamond, 2013). This is essential for promoting original and pragmatic ideas without formal support. When employees focus on core responsibilities within leadership-supported environments, their attentional focus becomes less vulnerable to peripheral distractions (Diamond, 2013; Forster and Lavie, 2009), thereby improving both the intensity and success likelihood of bootlegging innovation attempts through concentrated cognitive investment. Therefore, work attention forms the core cognitive bridge that transforms leader humility into employee bootlegging innovative behavior.

*H*2: Work attention mediates the relationship between leader humility and bootlegging innovative behavior.

### Mediating effect of thriving at work

Based on the literature, thriving at work—comprising both learning and vitality—plays a crucial mediating role between leader humility and bootlegging innovative behavior. Thriving reflects an employee’s psychological state of continuous growth and energy at work (Spreitzer et al., 2005; Niessen et al., 2012). The coexistence of learning and vitality is essential for employees’ positive functioning in their roles (Porath et al., 2012). Motivation and a forward-looking orientation emerge from this combination, empowering individuals to pursue self-initiated innovation activities outside formal organizational boundaries (Criscuolo et al., 2014).

Prior studies have emphasized the significance of individual learning (Eldor and Harpaz, 2016), vitality (Carmeli and Spreitzer, 2009), and positive psychological states (Fredrickson, 2001) for innovative outcomes. While creativity primarily involves idea generation, bootlegging innovative behavior encompasses generating, adopting, and implementing novel yet useful ideas without formal authorization (Qu et al., 2023; Criscuolo et al., 2014). Though bootlegging offers potential benefits, employees are not always intrinsically motivated to engage in such risky, resource-demanding behaviors.

Drawing on the socially embedded model of thriving at work (Van den Broeck et al., 2016), employee’s perceived motivation is shaped by the social context in which they are embedded. Leader humility—characterized by openness to feedback, acknowledgment of others’ strengths, and awareness of personal limitations (Owens and Hekman, 2012)—creates a supportive environment that enhances employees’ sense of psychological safety and belonging (Carmeli et al., 2015). This, in turn, promotes vitality and learning, allowing employees to explore and engage in bootlegging innovative behavior despite its inherent risks. Leaders play a central role in channeling employees’ attention and resources toward challenging goals because they control key developmental opportunities and constitute an essential part of the employees’ social environment (Zhou and George, 2001). Humble leadership signals trust and empowerment, encouraging employees to take proactive steps toward innovation even outside formal structures (Owens et al., 2019).

From the perspective of self-determination theory (SDT; Deci and Ryan, 2000), thriving at work represents an intrinsically motivated state through which basic psychological needs for autonomy, competence, and relatedness are satisfied. Leader humility strengthens these needs by providing autonomy support and recognition (Rego et al., 2017). As employees experience greater psychological safety and vitality, they are more likely to allocate cognitive and emotional resources toward bootlegging innovative initiatives. The humble leader’s acknowledgment of employee contributions enhances competence, while relational humility fosters a sense of connectedness, both critical to sustaining intrinsic motivation (Van den Broeck et al., 2016).

Therefore, thriving at work functions as a psychological conduit through which leader humility translates into greater bootlegging innovative behavior. In such con-texts, leader humility operates as an external motivational source, while thriving at work embodies employees’ internalized drive for learning, exploration, and innovation. Together, this dual mechanism enables employees to flourish under humble leadership, continuously generating and implementing novel ideas beyond formal organizational constraints.

Based on the above, we hypothesize:

*H*3: Thriving at work mediates the relationship between leader humility and bootlegging innovative behavior.

### The chained mediation role of work attention and thriving at work

Leader humility exerts a significant influence on employees’ work concentration, encompassing task engagement and attentional focus, as humble leaders actively acknowledge knowledge limitations to dismantle traditional hierarchical authority (Owens et al., 2013; Wang et al., 2018). Their non-defensive communication patterns reduce employees’ anxiety about negative evaluations and mitigate organizational defensiveness, fostering psychological safety that enables sustained attention on core tasks (Iqbal et al., 2020). Within this open dialogic environment shaped by leader humility, employees process work-related information more efficiently, enhancing cognitive resource allocation for creative problem-solving rather than anxiety-driven error correction (Cheung et al., 2020). The resulting cognitive surplus allows employees to channel mental resources toward innovative thinking and task execution.

We believe that the shaping process of work attention on thriving at work is essentially a coupling process between the directional input of cognitive resources and the dynamic accumulation of psychological capital. From a cognitive neuroscience perspective, sustained work attention operates through the coupling of prefrontal cortex activation and anterior cingulate engagement, optimizing neural resource distribution (Ulrich et al., 2014). When attentional focus remains anchored on work objectives, suppressed activity in the default mode network reduces mental clutter, facilitating flow states where dopamine and norepinephrine interactions heighten task enjoyment (Ulrich et al., 2014). Ulrich et al. (2014) showed that individuals in the flow state were significantly more likely to assess task pleasure in the synergistic effect of dopamine and norepinephrine. This immersive experience not only directly enhances the vitality of thriving at work, but also continuously stimulates exploratory learning behaviors through a dynamic balance of cognitive challenges and skill levels. According to the theory of resource conservation, maintaining high-quality work attention constitutes a strategic investment behavior of psychological resources (Hobfoll, 1989; Usman et al., 2020). When employees are engaged in deep attention work for a longer period time, they are more efficient in learning skills, and self-efficacy and achievement motivation work together in this process, which promotes the development of employees’ thriving at work.

Thriving at work represents a positive state of mind, not only in terms of achievement in work outcomes, but also in terms of emotion, motivation, development, and other aspects (Spreitzer et al., 2005). Thriving at work is characterized by a high level of well-being and self-efficacy, as well as motivation for continuous personal development and learning (Spreitzer et al., 2005; Liu et al., 2021). Employees who achieve thriving at work can realize their potential and find the right direction to improve their behavior (Riaz et al., 2018). Additionally, thriving at work promotes positive emotions, growth, and fulfillment in individuals, positively influencing the intrinsic motivation of employees, who are actively engaged in their work, innovative in their work, and flexible in responding to challenges (Ye et al., 2020; Usman et al., 2020). This thriving state then catalyzes bootlegging innovative behavior. Bootlegging innovative behavior refers specifically to those secret innovation activities initiated by employees without formal approval of the organization.

According to previous research, thriving at work is often accompanied by an in-crease in employees’ self-confidence and self-efficacy, making them more willing to take risks and challenge themselves, thereby driving innovation (Usman et al., 2020). Such a heightened sense of confidence is essential for engaging in bootlegging innovative behavior, which is inherently characterized by high risk and uncertainty. It encourages employees to take unauthorized risks and to challenge themselves beyond formal boundaries. When employees are thriving at work, they experience a greater sense of psychological safety within the organization; even when experimenting with new approaches without formal support, they are less preoccupied with the possibility of failure (Iqbal et al., 2020). This sense of security and support helps them to boldly produce and implement innovative ideas (Ye et al., 2020). Additionally, thriving at work employees tend to be constantly learning and self-improving, which lays the foundation for bootlegging innovative behavior. Employees are more likely to acquire new expertise in the process of learning and growing, which is conducive to the generation and implementation of new ideas (Ye et al., 2020; Usman et al., 2020). By acquiring new knowledge and skills at work, employees become better equipped to identify and address problems that may go unnoticed or under-resourced by the organization. They are thus able to respond to dynamic environmental changes in swift and innovative—albeit often covert—ways (Nanyangwe et al., 2021).

In summary, this chain mediation process unfolds as follows: leader humility (Owens et al., 2013) first fosters a psychologically safe environment (Iqbal et al., 2020), allowing employees to shift their focus from interpersonal defense to task execution, thereby freeing and concentrating their work attention (Cheung et al., 2020). This sustained investment of cognitive resources (Hobfoll, 1989) transforms into experiences of flow and skill acquisition, which accumulate into the psychological capital of thriving at work, reflected in enhanced vitality, learning, and self-efficacy (Spreitzer et al., 2005; Liu et al., 2021). Ultimately, this thriving state—particularly the heightened confidence, willingness to take risks, and capacity for self-directed learning it engenders (Usman et al., 2020)—provides employees with the psychological resources and intrinsic motivation necessary to engage in bootlegging innovative behavior. In doing so, employees are empowered and motivated to pursue valuable innovation opportunities beyond formal organizational constraints (Ye et al., 2020).

Therefore, Hypothesis 4 is proposed:

*H*4: Work attention and thriving at work mediate the relationship between leader humility and bootlegging innovative behavior in a chained mediation model.

**Figure 1:**
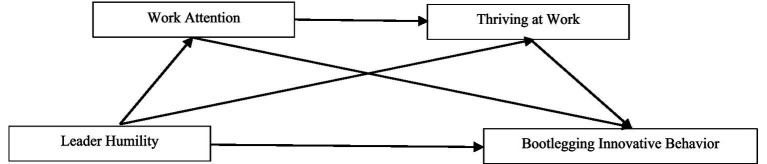
Research model.

The research model of this study is shown in Figure 1.

## Methods

SPSS, Stata, and M-plus were used for data analysis. M-plus was used for the confirmatory factor and path analyses.

### Measures

The questionnaire used a 5-point Likert scale, where 1 = strongly disagree, 2 = disagree, 3 = average, 4 = agree, and 5 = completely agree.

Leader humility is measured using six items from Owens et al. (2013). The scale consists of three dimensions: willingness to see the self accurately, appreciation of others’ strengths and contributions. Sample items include, “This person actively seeks feedback, even if it is critical”, “This person takes notice of others` strengths” and “This person is open to the ideas of others”, etc. (Cronbach’s alpha = 0.902).

Work attention was measured using four items from Rothbard (2001). Sample items include, “I spend a lot of time thinking about my work,” “I focus a great deal of attention on my work,” “I concentrate a lot on my work,” “I pay a lot of attention to my work” (Cronbach’s alpha = 0.854).

Thriving at work was measured using five items from Porath et al. (2012). The scale includes two dimensions: learning and vitality. Sample items include, “At work, I find myself learning often” and “I have energy and spirit,” etc. (Cronbach’s alpha = 0.885).

Bootlegging innovative behavior was measured using five items from Criscuolo et al. (2014). Sample items include, “I have the flexibility to work my way around my official work plan, digging into new potentially valuable business opportunities”, “I am running several pet projects that allow me to learn about new areas”, and “I proactively take time to work on unofficial projects to seed future official projects,” etc. (Cronbach’s alpha = 0.893).

### Participants and procedure

The survey subjects of this study were eight regional and national property companies from Henan, Jiangsu, and Zhejiang provinces in China. Before conducting the survey, we have fully explained the purpose and procedures of this survey to the participants, obtaining informed consent from all participants without any external pressure. We assure participants that all data is collected anonymously and is used only for academic research.

By measuring different variables at different times, the immediate errors commonly associated with cross-sectional studies are reduced (Podsakoff et al., 2003). We first collected demographic information, leader humility, and work attention data from participants (Time 1). At this stage, 533 valid questionnaires were obtained. Four weeks later (Time 2), participants who completed the Time 1 survey were surveyed and reported data on thriving at work and bootlegging innovative behavior. After the second round of data screening, we retained 427 valid data, with a valid response rate of 85%.

### Control variables

The gender, age, educational background, and tenure of respondents were used as control variables. Therefore, demographic variables such as gender, age, and educational background were used as control variables in the empirical analysis. Gender was encoded as 1 for males and 2 for females. Age was measured in years, and education was measured in the years in which the participant completed that stage of education.

## Results

### Descriptive statistics

Descriptive statistical analyses were performed for demographic variables based on data collected from 427 participants. Of the participants, 55.04% were males and 44.96% were females. In terms of age, participants aged under 30 years old accounted for the highest proportion at 54.56%, followed by participants aged 31–40, accounting for 20.14%; the participants aged over 51 constituted the lowest percentage at 7.26%. Regarding academic qualifications, the proportion of participants with a high school or technical secondary school and college education was the highest at 29.51%. The pro-portion of participants with a master’s degree was the lowest at 7.49%. Regarding tenure, 1–5 years accounted for the highest proportion (53.16%). Descriptive data are shown in Table 1.

**Table 1:** Descriptive analysis of participants.

Demographic variable	Type	Frequency	Ratio (%)
Gender	Male	235	55.04
	Female	192	44.96
Age (years)	Under 25	130	30.44
	26–30	103	24.12
	31–35	51	11.94
	36–40	35	8.20
	41–50	77	18.03
	Over 51	31	7.26
Major	Junior high school and below	36	8.43
	High school/technical secondary school	126	29.51
	College	126	29.51
	Bachelor’s degree	107	25.06
	Master’s degree	32	7.49
Tenure (years)	1–5	227	53.16
	6–10	56	13.11
	11–15	33	7.73
	16–20	54	12.65
	Over 21	57	13.35
Total		427	100.00

### Confirmatory factor analysis and reliability analysis

Confirmatory factor analysis was used to test the model fit. The results were as follows: χ^2^ (p) = 222.859 (0.002), χ^2^/df = 1.359, RMSEA = 0.029, CFI = 0.988, TLI = 0.987, SRMR = 0.027. The fitting index of the model showed a good effect. Although the chi-squared test results (χ^2^ = 222.859, *p* = 0.002) illustrated a significant difference in model fitting, the chi-squared/degrees-of-freedom ratio (χ^2^/df = 1. 359) indicated that it was acceptable, considering the effect of large samples. RMSEA values less than 0.05 reflect a small approximation error, while those between 0.05 and 0.08 reflect an acceptable approximation error, and values greater than 0.10 reflect a poor model fit (Cudeck and Browne, 1992). The RMSEA value was 0.029, which was lower than 0.05, indicating that the model fit of data was good. The CFI and TLI values were 0.988 and 0.987, respectively. Both were greater than 0.90, indicating that the model had a high degree of fit. Additionally, the SRMR value was 0.027, which was less than 0.08, further verifying that the model fit was good.

The average variance extraction (AVE) and composite reliability (CR) were analyzed. The AVE value measures the proportion of explanatory variance for each indicator in the construct (Hair Jr et al., 2017), and the values for each variable were as follows: 0.607 for leader humility, 0.595 for work attention, 0.605 for thriving at work, and 0.629 for bootlegging innovative behavior. All values were greater than 0.5, indicating good convergent validity.

The CR value measures the internal consistency of the construct (Sen, 1993). The CR values for each variable were as follows: 0.902 for leader humility, 0.854 for work attention, 0.884 for thriving at work, and 0.894 for bootlegging innovative behavior. All values were greater than 0.7, indicating good confidence.

A reliability analysis was used to assess the agreement between items in a questionnaire or scale (Tavakol and Dennick, 2011). The Cronbach’s alpha values calculated in this study were as follows: leader humility was 0.902, work attention was 0.854, thriving at work was 0.855, and bootlegging innovative behavior was 0.893, all of which were greater than 0.80. This indicated a high degree of agreement between the scale items. The results are summarized in Table 2.

**Table 2:** Confirmatory factor analysis and reliability analysis.

Variables	Items	Estimate	SE	AVE	CR	Cronbach’s alpha
Leader humility	LH1	0.770	0.022	0.607	0.902	0.902
	LH2	0.745	0.024			
	LH3	0.784	0.021			
	LH4	0.766	0.023			
	LH5	0.750	0.024			
	LH6	0.855	0.016			
Work attention	WA1	0.741	0.027	0.595	0.854	0.854
	WA2	0.751	0.026			
	WA3	0.775	0.025			
	WA4	0.817	0.022			
Thriving at work	TW1	0.730	0.026	0.605	0.884	0.885
	TW2	0.767	0.023			
	TW3	0.740	0.025			
	TW4	0.794	0.021			
	TW5	0.853	0.017			
Bootlegging innovative behavior	BIB1	0.816	0.019	0.629	0.894	0.893
	BIB2	0.686	0.028			
	BIB3	0.755	0.023			
	BIB4	0.833	0.018			
	BIB5	0.862	0.016			
Model fit index	χ^2^(p) = 222.859 (0.002), χ^2^/df = 1.359, RMSEA = 0.029, CFI = 0.988, TLI = 0.987, SRMR = 0.027					

*N =* 427. LH, leader humility; WA, work attention; TW, thriving at work; BIB, bootlegging innovative behavior.

### Common method biases

To assess the potential impact of common method bias (CMB), this study employed Harman’s single-factor test. The results indicate that the first factor accounted for 45.84% of the total variance. Since the variance explained by the first factor did not exceed 50%, and no single factor emerged that loaded on all variables, the findings suggest that common method bias is not a serious concern in this study. Although this result implies that potential CMB risks should still be acknowledged, it does not provide sufficient evidence to conclude that CMB substantially affects the study’s outcomes, consistent with the perspective of Podsakoff et al. (2003).

### Correlation analysis

The mean values for leader humility, work attention, thriving at work and boot-legging innovative behavior were 3.714, 3.803, 3.748 and 3.708, respectively. The standard deviations (SD) of leader humility, work attention, thriving at work and bootlegging innovative behavior were 0.791, 0.759, 0.793 and 0.785, respectively. Leader humility was associated with work attention (*r* = 0.422, *p* < 0.001), thriving at work (*r* = 0.646, *p* < 0.001) and bootlegging innovative behavior (*r* = 0.614, *p* < 0.001). Work attention was positively correlated with thriving at work (*r* = 0.374, *p* < 0.001) and bootlegging innovative behavior (*r* = 0.461, *p* < 0.001). Thriving at work was positively correlated with bootlegging innovative behavior (*r* = 0.640, *p* < 0.001). The square root of AVE of all variables is greater than the absolute value of the correlation coefficient between this variable and other variables, so it is considered that there is good discriminant validity between each variable (Fornell and Larcker, 1981). The results are summarized in Table 3. The results of the variable correlation test meet the prerequisites of regression analysis.

**Table 3:** Correlation analysis.

Variables	Mean	SD	Gender	Age	EB	T	LH	WA	TW	BIB
G	1.450	0.498	1							
Age	2.810	1.699	0.040	1						
EB	2.937	1.087	0.105**	0.085*	1					
T	2.199	1.511	−0.019	0.881***	−0.060	1				
LH	3.714	0.791	0.024	0.050	0.090*	0.034	0.779			
WA	3.803	0.759	0.074	0.091*	0.083*	0.075	0.422***	0.771		
W	3.748	0.793	0.096**	0.044	0.058	0.044	0.646***	0.374***	0.778	
BIB	3.708	0.785	−0.005	−0.026	0.098**	−0.002	0.614***	0.461***	0.640***	0.793

*N* = 427. Diagonal is the square root of AVE. ***p < 0.001, **p < 0.01, *p < 0.05. G, gender; EB, educational background; T, tenure; LH, leader humility; WA, work attention; TW, thriving at work; BIB, bootlegging innovative behavior.

### Hypothesis test

The regression analysis results showed that leader humility had a significant direct positive effect on bootlegging innovative behavior (*β* = 0.283, *p* < 0.001), and Hypothesis 1 was supported. Leader humility had a significant direct positive effect on work attention (*β* = 0.397, *p* < 0.001) and thriving at work (*β* = 0.645, *p* < 0.001), work attention had a significant direct positive effect on thriving at work(*β* = 0.124, *p* < 0.001) and bootlegging innovative behavior (*β* = 0.260, *p* < 0.001), and thriving at work had a significant direct positive effect on bootlegging innovative behavior (*β* = 0.386, *p* < 0.001). Therefore, the next step can be carried out to test the mediating effects of work attention and thriving at work and the chain mediating effect. The results are summarized in Table 4.

**Table 4:** Results of regression analysis.

Items	WA		TW			BIB			
	M1	M2	M3	M4	M5	M6	M7	M8	M9
G	0.099	0.090	0.148	0.134*	0.123*	−0.011	−0.025	−0.048	−0.095
Age	0.023	0.016	−0.015	−0.024	−0.026	−0.085	−0.094*	−0.098**	−0.088*
EB	0.052	0.027	0.040	−0.000	−0.003	0.090*	0.052	0.045	0.046
T	0.018	0.016	0.040	0.036	0.034	0.087	0.083	0.079	0.066
LH		0.397***		0.645***	0.596***		0.608***	0.505***	0.275***
WA					0.124**			0.260***	0.212***
TW									0.386***
R^2^	0.018	0.019	0.014	0.425	0.436	0.017	0.388	0.440	0.526
△R^2^	−0.009	−0.010	−0.009	−0.007	−0.008	−0.009	−0.003	−0.008	−0.008
F	1.95 (4, 422)	19.45(5, 421) ***	1.51(4, 422)	62.16(5, 421) ***	54.14(6, 420) ***	1.80(4, 422)	53.48(5, 421) ***	54.94(6, 420) ***	66.29(7, 419) ***

*N =* 427. ***p < 0.001, **p < 0.01, *p < 0.05. G, gender; EB, educational background; T, tenure; LH, leader humility; WA, work attention; TW, thriving at work; BIB, bootlegging innovative behavior.

The M-plus serial multiple mediation model was used to examine how the variables used in this study interacted. To test the indirect effects of the mediation and sequence mediation hypotheses, the bootstrap method was used to perform an analysis using the M-plus statistical analysis software. If the upper and lower bounds of the coefficients obtained in the middle of the 95% confidence interval (IC) do not include “0,” they can be determined as significant values (Lee et al., 2022). Table 5 lists the results of the path analysis. The results of the bootstrap method showed that leader humility → work attention → bootlegging innovative behavior. There was a positive mediating effect on innovative behavior (*β* = 0.083, CI: 0.047–0.131), and Hypothesis 2 was supported. The positive mediating effect of leader humility → thriving at work → bootlegging innovative behavior (*β* = 0.226, CI: 0.162–0.299) was also confirmed, supporting Hypothesis 3. In addition, the sequential continuous positive mediating effect of leader humility → work attention → thriving at work → bootlegging innovative behavior (*β* = 0.020, CI: 0.005–0.044) did not include 0 at the 95% confidence level. Work attention and thriving at work mediate the relationship between leader humility and bootlegging innovative behavior. Leader humility adds work attention, thriving at work, and thriving at work adds bootlegging innovative behavior, Hypothesis 4 is supported. The results are summarized in Tables 5, 6.

**Table 5:** Path analysis.

Path	Estimate	S. E.	t	*p*	LLCI	ULCI
Leader humility → Work attention	0.422	0.053	8.024	0.000	0.314	0.518
Leader humility → Thriving at work	0.594	0.049	12.050	0.000	0.490	0.683
Leader humility → Bootlegging innovative behavior	0.283	0.063	4.513	0.000	0.167	0.412
Work attention → Thriving at work	0.124	0.047	2.609	0.009	0.033	0.219
Work attention →Bootlegging innovative behavior	0.198	0.042	4.757	0.000	0.119	0.280
Thriving at work →Bootlegging innovative behavior	0.383	0.060	6.387	0.000	0.261	0.492

**Table 6:** Bootstrap indirect effect test.

Path	Effect	Boot SE	Boot LLCI	Boot ULCI
Leader humility → Work attention →Bootlegging innovative behavior	0.083	0.021	0.047	0.131
Leader humility → Thriving at work → Bootlegging innovative behavior	0.226	0.035	0.162	0.299
Leader humility → Work attention→ Thriving at work → Bootlegging innovative behavior	0.020	0.009	0.005	0.044
Total	0.609	0.052	0.502	0.702

*N =* 427. The indirect effect estimated was tested for significance using 95% bi-as-corrected, bootstrapped confidence intervals. Bootstrap resampling = 5,000.

## Conclusion

### Results and discussion

This study addresses a core question raised in the introduction. Why do employees engage in informal innovation without formal authorization and with clear risk? The findings show that such behavior is not driven only by individual risk preference. It is embedded in cognitive and psychological conditions shaped by leadership. Leader humility directs employees’ attention toward meaningful work tasks. It also supports a state of thriving at work. Together, these conditions increase the likelihood of constructive bootlegging innovative behavior.

This study examines employees’ behavior from the perspective of self-development, focusing on the antecedents and influencing factors of bootlegging innovative behavior—a form of self-initiated, unauthorized innovation that plays a crucial role in sustaining an organization’s adaptability and competitive advantage (Criscuolo et al., 2014). Specifically, this research investigates the relationship between leader humility and employees’ bootlegging innovative behavior, as well as the chained mediating effects of work attention and thriving at work, using data from employees in China’s manufacturing and service industries. The empirical findings provide strong support for the proposed hypotheses. Leader humility exerts a significant direct positive influence on bootlegging innovative behavior, while both work attention and thriving at work also have significant direct positive effects. Moreover, the chained mediation effect of work attention and thriving at work in the relationship between leader humility and bootlegging innovative behavior is empirically validated. In other words, leader humility not only directly promotes employees’ engagement in bootlegging innovation but also indirectly facilitates it by enhancing their focused attention and psychological thriving at work. By explicitly addressing the initial question of why employees engage in risky and informal innovation, this study clarifies how leader humility shapes the cognitive and psychological conditions that make such behavior possible.

Humble leaders serve as role models who create motivational and supportive work environments. By recognizing employees’ contributions and showing openness to feedback, humble leaders enhance employees’ sense of psychological safety and intrinsic motivation (Owens and Hekman, 2012; Rego et al., 2017). As a result, employees experience greater vitality and competence, devoting more cognitive resources to their work tasks rather than organizational politics. This heightened attentional engagement fosters flow and learning, which accumulate into thriving at work—a state characterized by vitality, learning, and self-efficacy (Spreitzer et al., 2005; Niessen et al., 2012). Employees who thrive at work are more likely to take the initiative to engage in creative, self-directed innovation, even in the absence of formal authorization, thereby exhibiting bootlegging innovative behavior.

Work attention and thriving at work are conceptually related. They reflect different psychological processes. Work attention is a cognitive process. It reflects the focused allocation of limited mental resources to work tasks. Thriving at work is a broader psychological state. It develops over time. It is characterized by vitality and learning. In the present model, work attention operates as an immediate cognitive mechanism. Thriving at work reflects a more long-term psychological outcome. Acknowledging their conceptual proximity does not weaken the mediation logic. It highlights functional and temporal differences between cognitive focus and psychological growth.

Grounded in self-determination theory (SDT; Deci and Ryan, 2000), this study posits that leader humility, work attention, and thriving at work jointly provide both extrinsic and intrinsic motivational foundations for employees’ bootlegging innovative behavior. The external support, encouragement, and recognition conveyed by humble leaders satisfy employees’ basic psychological needs for autonomy, competence, and relatedness (Van den Broeck et al., 2016), while thriving at work transforms these experiences into intrinsic motivation that drives covert innovation efforts. Consequently, employees are more inclined to pursue unapproved yet valuable innovation opportunities, contributing to both personal growth and organizational advancement.

This research offers a novel explanation for the mechanism through which leader humility influences employees’ bootlegging innovative behavior and introduces a fresh perspective on the roles of work attention and thriving at work. By incorporating these two constructs as sequential mediators, this study expands the theoretical under-standing of leadership and employee innovation processes. The findings hold both theoretical and practical significance: theoretically, they deepen insights into how humble leadership cultivates psychological and cognitive conditions conducive to unauthorized innovation; practically, they highlight the importance of fostering humility and thriving as strategic levers for encouraging responsible, bottom-up innovation in organizations.

### Theoretical and practical implications

This study extends existing research on the influence of external and internal factors on employee behavior by hypothesizing and empirically verifying the chained mediating role of work attention and thriving at work in the relationship between leader humility and bootlegging innovative behavior. Prior research on leader humility, thriving at work, and employee behavior remains relatively limited within psychology and organizational behavior domains (Goh et al., 2022). By examining this relationship from the perspective of individual self-development, the present study offers a novel explanation of how leader humility shapes employees’ engagement in bootlegging innovative behavior and enriches the broader literature on leadership and employee behavioral outcomes. The findings suggest that work attention and thriving at work together provide a meaningful mechanism through which leader humility facilitates bootlegging innovation. Drawing on conservation of resources theory (Hobfoll, 1989) and self-determination theory (Deci and Ryan, 2000), this study further analyzes how external and internal motivational drivers interact to promote thriving at work, thereby deepening theoretical understanding of both constructs.

Previous studies have examined various pathways linking leader humility and employee innovation from distinct perspectives. For example, Zhou and Wu (2018) explored how leader humility influences innovative behavior through employees’ core self-evaluations within a leader–employee interaction framework, while Abbas and Wu (2021) demonstrated that in collectivist cultures, humble leadership enhances organizational justice, thereby stimulating innovation. Building on these insights, the present research investigates how work attention and thriving at work jointly mediate the influence of leader humility on bootlegging innovative behavior through a serial mediation model. Accordingly, this study contributes to the understanding of how leader humility fosters employees’ self-directed, unauthorized innovation and provides a fresh perspective on its underlying psychological and cognitive mechanisms.

Bootlegging innovative behavior, though informal and unapproved, is often a critical source of organizational creativity and adaptability (Criscuolo et al., 2014). To meet the demands of dynamic and diverse markets, employees must possess not only innovative capacity but also the autonomy and confidence to act on new ideas outside formal channels. Therefore, organizations can nurture an innovation-driven culture that aligns personal growth with organizational development by enhancing leader humility and fostering a psychologically supportive work environment that stimulates employees’ intrinsic motivation. As leader humility strengthens employees’ work attention and thriving at work—both of which indirectly promote bootlegging innovative behavior—organizations can integrate humility cultivation into leadership development programs. Encouraging leaders to acknowledge their limitations, value employees’ contributions, and maintain openness to learning can create a more inclusive and empowering work climate, ultimately driving novel and valuable innovations within the organization.

Leader humility can serve as an important signal. It legitimizes learning, experimentation, and voice. These factors are critical for constructive bootlegging innovative behavior. Humility alone does not ensure positive outcomes. Organizations need to balance humility with appropriate control. Employees should have autonomy to explore informal innovation. Clear boundaries remain necessary. Ethical guidelines matter. Shared goals also matter. These elements help prevent dysfunctional or high-risk bootlegging. In practice, managers can achieve this balance by combining humble leadership with clear expectations about risk and resource use. Open communication should be encouraged. Employee contributions should be recognized. Reasonable experimentation should be tolerated. Regular reflection helps. Boundary setting helps. Feedback mechanisms help. In this way, organizations can unlock the creative potential of informal innovation. They can also maintain alignment with organizational goals.

Moreover, the positive mediating roles of work attention and thriving at work imply that organizations should actively implement practices that enhance these states among employees. For instance, companies can develop career growth programs, pro-vide learning resources, and create opportunities for skill development to sustain employees’ motivation and long-term growth. Additionally, attention to employees’ physical and psychological well-being—through flexible work arrangements, psycho-logical counseling, and work–life balance initiatives—can foster thriving at work and enhance employees’ energy and engagement. When employees experience sustained vitality and learning, they are more likely to engage in self-initiated, covert innovation efforts that benefit both individual and organizational development.

In summary, this research contributes to leadership and innovation literature by revealing how leader humility indirectly promotes bootlegging innovative behavior through the combined mediating effects of work attention and thriving at work. These findings not only advance theoretical understanding of humble leadership’s motivational mechanisms but also offer practical guidance for organizations seeking to encourage responsible, bottom-up innovation through psychologically supportive and autonomy-enhancing work environments.

### Limitations and future research

Despite several strengths, this study has limitations. First, it relies mainly on employee self-reported data. This reliance may raise concerns about subjective bias and common method variance. A two-wave design was used to separate key variables over time. This approach helps reduce such risks. Future research can further strengthen causal inference. Multi-source data can be included. Supervisor or peer ratings are useful. More objective or behavioral indicators of innovation may also help.

The sample primarily comprises employees from China’s manufacturing and service sectors, which may restrict the generalizability of the findings to other industries or cultural contexts. Given the relatively higher power distance and strong authority norms in the Chinese cultural context, leader humility may carry distinct symbolic meaning. In such contexts, humble leadership can signal a stronger deviation from hierarchical expectations and may therefore exert more salient effects on employees’ psychological safety and initiative. Cross-cultural research suggests that humility is interpreted and enacted differently across Eastern and Western contexts (Oc et al., 2015; Kim and Kim, 2025; Ghamrawi et al., 2025). Accordingly, future studies should examine whether the proposed model holds in low power-distance cultures, where leader humility may be perceived as normative rather than exceptional. Employees’ leadership preferences, motivation for innovation, work attention, and thriving at work experiences may vary substantially across cultural settings. Moreover, the focus on specific industries may limit the applicability of findings regarding leader humility and bootlegging innovative behavior in other contexts. Compared with industries such as technology or education, the manufacturing and service sectors may differ in how they define, encourage, and manage bootlegging innovation. Future research should examine the model in diverse cultural and industrial contexts to investigate how cultural norms and industry characteristics influence the relationship among leader humility, work attention, thriving at work, and bootlegging innovative behavior.

Although work attention and thriving at work are treated as distinct constructs in the theoretical model, they are conceptually related. Work attention emphasizes the focused allocation of cognitive resources to tasks. Thriving at work reflects a broader psychological state. It is characterized by vitality and learning. This conceptual proximity offers an important opportunity for future research. Longitudinal designs may be useful. Experience sampling methods may also help. These approaches can examine how momentary cognitive focus accumulates into sustained psychological growth.

The influence of leader humility on work attention and thriving at work may fluctuate across different career stages. Future research could address this limitation by employing an alternative longitudinal research design to explore how these variables interact and evolve over career development stages. For instance, leader humility might exert a stronger impact on employees’ work attention and thriving at work during the initial stages of organizational entry, when psychological safety and support are most crucial, while this influence may weaken or transform as employees gain autonomy and experience. Longitudinal approaches may also reveal how personal traits such as grit moderate the relationship between thriving and bootlegging innovative behavior throughout employees’ career trajectories. In addition, a longitudinal design combined with experimental methods may offer stronger causal tests. Scenario-based experiments can help examine the causal links between leader humility and employees’ psychological and behavioral outcomes.

Although this study contributes valuable insights into the mechanism linking leader humility and bootlegging innovative behavior through work attention and thriving at work, future research should expand the methodological scope and contextual diversity. Future research adopting a behavioral perspective on leadership humility could further enhance both theoretical precision and practical applicability, particularly in leadership development and training contexts.

Although leader humility in the present study is measured through employees’ perceptions, this approach does not preclude the relevance of alternative methodological perspectives. Perceptual measures capture employee’s lived experiences and subjective interpretations, which are critical for understanding motivational and behavioral outcomes (Owens et al., 2013; Wang et al., 2018). At the same time, recent research suggests that leader humility can also be examined through observable verbal and nonverbal behaviors, such as language use, vocal tone, facial expressions, and body posture (Signorello et al., 2012; D’Errico and Poggi, 2019). Recognizing these complementary approaches does not diminish the robustness of perception-based measurement. Instead, it highlights the multi-level and multi-modal nature of leader humility. Future research can combine perceptual measures with behavioral coding and computational methods. Such approaches can capture the micro-communicative mechanisms of leader humility with greater precision. Importantly, acknowledging alternative methodological approaches does not weaken the current empirical strategy, but rather situates it within a broader and more integrative research agenda on leader humility.

## Data Availability

The original contributions presented in the study are included in the article/supplementary material, further inquiries can be directed to the corresponding author.
